# Advances in Understanding the Mechanisms of Treatment for Gouty Arthritis: A Comprehensive Review

**DOI:** 10.33549/physiolres.935631

**Published:** 2025-10-01

**Authors:** Sisi CHEN, Zeguang LI, Xiaowei DENG, Lijuan GAO

**Affiliations:** 1 Department of Rheumatology, First Affiliated Hospital, Heilongjiang University of Chinese Medicine, Harbin, China; 2 Fifth Cardiovascular Department, First Affiliated Hospital, Heilongjiang University of Chinese Medicine, Harbin, China

**Keywords:** Gouty arthritis, Serum urate, Hyperuricemia, Crystal deposition, Colchicine

## Abstract

Gouty arthritis is a type of inflammatory arthritis that is mediated by the deposition of monosodium urate crystals and is an important burden on healthcare worldwide. The aim of this comprehensive review is to discuss the most recent advances regarding the mechanisms of treatment for gout, from classic pharmacological interventions to emerging therapeutic strategies. The chapter dissects the pathophysiology of gout through hyperuricemia, crystal deposition, and inflammatory responses to form a basis for the discussion of current treatment approaches; pharmacological interventions are described-side by side with lifestyle modifications-including NSAIDs, colchicine, and xanthine oxidase inhibitors. Newer approaches to management are discussed, including the use of biologics targeting IL-1β, newer agents in development, and personalized medicine. It also outlines the future directions in gout research, focusing on the development of novel imaging techniques, biomarkers for treatment response, and targeting novel pathways. This review serves as an overall guide for clinicians and researchers and all other stakeholders interested in further advancing the specialty of gouty arthritis.

## Introduction

Gouty arthritis, a form of inflammatory arthritis, has consistently attached its importance in the annals of medical history, tracing roots to the deposition of monosodium urate crystals within the joints [1]. Characterized by recurrent bouts of excruciating pain, redness, and swelling, gout affects a substantial proportion of the global population, imposing a considerable burden on affected individuals and healthcare systems alike [2]. Epidemiological studies disclose that the prevalence rate for gout is increasing, and this increase has been attributed to dietary changes, sedentary lifestyles, and increasing age [3].

Gout is the most prevalent form of inflammatory arthritis worldwide, affecting an estimated 41 million adults globally, with incidence and prevalence both rising over the past two decades [3]. The burden of disease includes not only joint pain and disability but also increased risk of cardiovascular, renal, and metabolic comorbidities [4]. Projections indicate that the prevalence of gout may continue to rise due to aging populations and increasing rates of obesity and metabolic syndrome.

Understanding the complex mechanisms leading to the treatment of gouty arthritis will be of paramount importance in order to reduce the impact of this disease on affected individuals [5]. Whereas conventional therapies have been efficacious, new research developments have been able to reveal novel insights into the molecular pathways that underpin the disease and response to its treatment. With these developments, a comprehensive review is essential in synthesizing and disseminating the evolving gouty arthritis therapeutics landscape.

This review highlights recent updates on the understanding of the mechanisms of treatment for gouty arthritis. A critical analysis of the recent literature is performed with the goal of providing a complex understanding of the pathophysiology of the condition, intricacies in current treatment modalities, and emergent strategies that will change the face of the therapeutic paradigm. In this regard, we attempt to add to the literature available that shapes clinical practice and secures better patient outcomes, as well as opens up new perspectives and opportunities for future advancements in the management of gouty arthritis.

The primary objective of this review is to synthesize recent advances in the mechanisms and management of gout, with a particular focus on emerging therapeutic strategies. In order to provide context for these developments, we also discuss relevant aspects of pathophysiology, biomarkers, and imaging, thereby offering an integrated perspective on modern gout care.

## Pathophysiology of Gouty Arthritis

**Fig. 1:**
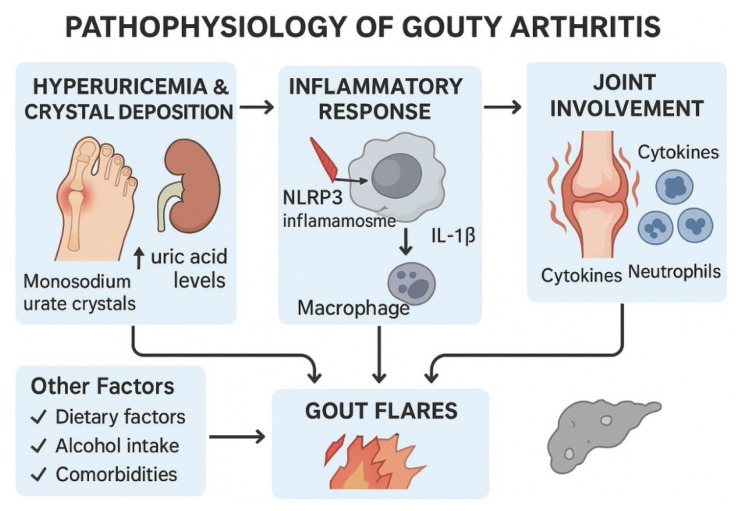
Schematic representation pathophysiology of gouty arthritis.

Gouty arthritis is a very complex and multifactorial disorder that develops as a consequence of a series of pathophysiological events related to each other, leading to the onset and further development of the disease [6]. The schematic representation pathophysiology of gouty arthritis has been given in Figure 1.

### Hyperuricemia and crystal deposition

Hyperuricemia is defined by elevated serum levels of serum urate, a condition that serves as the foundation of gout pathogenesis [7]. Under conditions of serum urate supersaturation, monosodium urate crystals precipitate in the joints, with a particular predilection for peripheral areas such as the big toe and fingers [8]. The pathogenesis involves both increased production and decreased renal excretion of serum urate, exacerbated by dietary factors (e.g., purine-rich foods and high alcohol intake) and genetic predisposition [8] Recent classification systems emphasize that urate homeostasis is influenced not only by renal excretion but also significantly by intestinal elimination – accounting for at least one-third of total urate excretion. Accordingly, the traditional “overproduction type” of hyperuricemia has been relabeled as ‘renal overload type,’ which encompasses both urate overproduction and extra-renal underexcretion (e.g., reduced intestinal elimination due to ABCG2 dysfunction). Genetic variants affecting renal and extrarenal urate transporters, such as URAT1, SLC2A9, and ABCG2, play a critical role by determining individual susceptibility to hyperuricemia and subsequent gout through their effects on serum urate reabsorption and excretion [9–12].

The formation of monosodium urate crystals serves as a trigger for inflammatory responses in gouty arthritis [13]. These needle-shaped crystals function as damage-associated molecular patterns (DAMPs), which activate the NLRP3 inflammasome in macrophages upon phagocytosis. This activation is pivotal for the maturation and secretion of pro-inflammatory cytokines, notably interleukin-1 beta (IL-1β) [14]. The release of IL-1β amplifies the inflammatory response, driving further recruitment of immune cells and perpetuating the cycle of inflammation. The resulting synovial inflammation and tissue damage are integral to the symptoms associated with gout flares, including acute pain, redness, and swelling of affected joints [15].

### Inflammatory response and joint involvement

The presence of monosodium urate crystals in the synovial fluid initiates a robust inflammatory response, which is a quintessential feature of gouty arthritis [16]. Chemotactic signals released from the crystals direct neutrophils to the site of deposition. Following their activation through the phagocytosis of crystals, neutrophils release a plethora of inflammatory mediators, including cytokines (such as interleukin-6 (IL-6), tumor necrosis factor-alpha (TNF-α)), chemokines, and prostaglandins [17]. This inflammatory cascade leads to synovial inflammation characterized by joint edema, increased warmth, and erythema.

The role of cytokines in perpetuating inflammation cannot be overstated. For example, cytokines like IL-6 and TNF-α play significant roles in sustaining inflammation and promoting further infiltration of neutrophils and macrophages [18]. Additionally, the activation of matrix metalloproteinases, stimulated by inflammatory mediators, contributes to ongoing joint damage and further exacerbates the clinical features associated with acute gout flares [19].

### Factors contributing to gout flares

Gout flares, characterized by sudden and severe flares of pain, epitomize the intermittent nature of the disease. Many factors give rise to the onset of gout flares; both internal and external factors contribute to this [20]. They range from serum urate diuresis, dietary factors here including purine-rich foods, intake of alcohol, and other comorbid diseases like hypertension and renal impairment [21]. The interplay of these elements further underlines the complex nature of gouty arthritis, whereby the fragile balance of serum urate metabolism and external influences can tip the scales toward acute exacerbation.

These processes interrelate in the unraveling of the pathophysiology of gouty arthritis and delineate potential points of therapeutic target and intervention. The succeeding review of evolving treatment modalities in the management of gout is based on a comprehensive understanding of the above mechanisms.

## Current treatment approaches

Gouty arthritis is a condition typified by frequent disabling flares; hence, its treatment is quite complex, being multilevel in approach [22]. The management currently involves pharmacologic intervention and lifestyle modifications. Both, however, remain important in the management of this inflammatory arthropathy (Table 1).

### Pharmacological interventions

Nonsteroidal Anti-Inflammatory Drugs (NSAIDs): NSAIDs, such as indomethacin and naproxen, constitute a cornerstone in the acute management of gout flares [23]. By inhibiting cyclooxygenase enzymes, NSAIDs mitigate pain and inflammation, offering rapid relief during acute episodes [24].

Colchicine: Colchicine has been known since ancient times and finds a modern application in inhibiting microtubule assembly. It is widely used both in the acute and prophylactic managements of gout, being effective in suppressing the inflammatory response given rise to by crystal deposition [25].

Corticosteroids: Intravenous or intra-articular corticosteroids offer powerful anti-inflammatory responses, thereby rapidly relieving pain and inflammation during acute gout flares. Systemic corticosteroids can be used in cases where other agents are contraindicated or poorly tolerated [26].

Xanthine Oxidase Inhibitors (e.g., Allopurinol, Febuxostat): They inhibit xanthine oxidase and consequently decrease the serum urate production at its production site. Allopurinol and febuxostat are widely indicated for long-term treatment and prevention of gout flares [27].

Uricosuric Agents (e.g., Probenecid): These drugs increase the renal excretion of serum urate, leading to reduced serum levels. Probenecid represents a typical example in this group of drugs and is generally prescribed in conditions with undersecretion of serum urate [28].

### Lifestyle modifications

Dietary Changes: Traditional dietary recommendations for gout have focused on limiting intake of purine-rich foods – such as organ meats, certain seafood, and beer – to reduce serum urate levels [29]. While it remains important for individuals with gout to recognize and avoid foods that may specifically trigger their flares, emerging evidence indicates that the overall dietary pattern may be more beneficial than strict purine restriction alone.

Recent large-scale studies and expert guidelines now favor the adoption of cardiometabolically healthy dietary patterns such as the Dietary Approaches to Stop Hypertension (DASH) diet and the Alternative Healthy Eating Index (AHEI), which are associated with lower serum urate levels, reduced risk of gout, and improvement in comorbidities like hypertension and insulin resistance [30,31]. The Mediterranean diet, which emphasizes fruit, vegetables, whole grains, healthy fats, and moderate dairy intake, has also been suggested as beneficial in observational studies [32].

Such dietary approaches confer additional anti-inflammatory effects and metabolic benefits beyond urate lowering, including improved glycemic control and cardiovascular risk profiles. Therefore, increasing focus is now placed on adopting healthy, balanced eating patterns that are sustainable and tailored to the individual, rather than prescribing strict, low-purine diets alone.

Hydration: In view of gouty flares, hydration should be adequate. More the intake of fluid, more the serum urate excretion, more dilute the blood, and hence less chances of depositing crystals [33].

Weight management: Obesity is considered a known risk factor for gout due to its relation with increased serum urate production and reduced excretion. Therefore, weight management strategies involve a balanced diet and regular exercise, contributing toward overall gout control [9].

Alcohol consumption: Avoidance of alcohol, particularly beer and spirits, is a key aspect of dietary management in gout. Alcohol increases serum urate levels by promoting the production of serum urate and inhibiting its renal excretion. Epidemiological studies have consistently shown that alcohol consumption – especially beer and spirits – is strongly associated with both the development of hyperuricemia and the incidence of acute gout flares. Wine appears to have a lesser effect, but even moderate intake may increase gout risk in susceptible individuals [32]. Clinical guidelines therefore recommend minimizing or eliminating alcohol intake, particularly during periods of frequent gout flares or uncontrolled hyperuricemia, as part of a comprehensive lifestyle approach to gout prevention and management [34].

## Therapeutic strategies and mechanisms of action in Gout

**Fig. 2:**
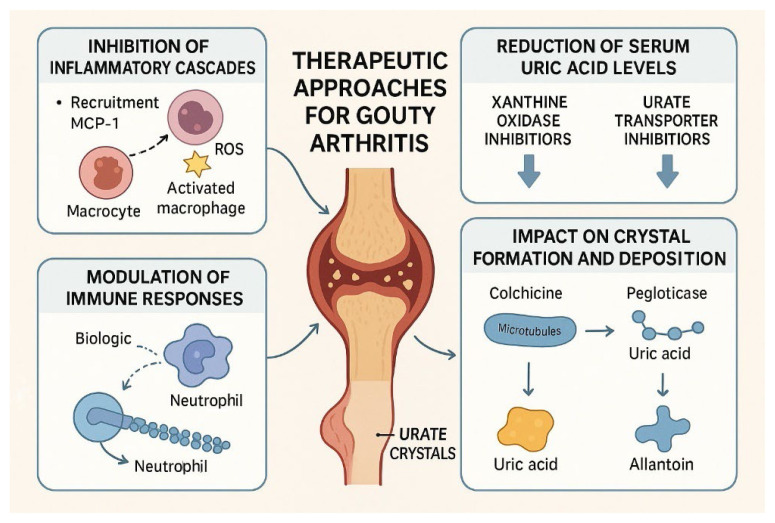
Therapeutic approaches to treat gouty arthritis.

Recent advancements in the understanding of gout’s pathophysiology have led to the development of a range of emerging therapeutic strategies aimed at providing more specific and effective treatment options for this complex inflammatory disease. These new approaches can complement traditional methods, thereby offering patients a more comprehensive treatment strategy (Fig. 2, Table 2).

### Urate-lowering therapies: Conventional agents and mechanisms

#### Xanthine oxidase inhibitors (XOIs)

Allopurinol and febuxostat are first-line agents that inhibit xanthine oxidase, the enzyme responsible for converting hypoxanthine and xanthine into uric acid, thereby reducing urate production and serum concentrations. Febuxostat offers an alternative for patients with mild-to-moderate chronic kidney disease, though long-term cardiovascular safety remains under evaluation. Clinical trials have confirmed their efficacy in maintaining serum urate targets and preventing flare recurrence. Dose titration and renal adjustment may be necessary to optimize efficacy and minimize adverse effects, especially in patients with CKD [48,49].

#### Uricosurics

Probenecid and lesinurad act by inhibiting renal tubular reabsorption of uric acid, primarily through blockade of the URAT1 transporter, increasing urinary excretion. These agents are appropriate adjuncts for patients with underexcretion of urate or inadequate response to XOIs, but increased risk of nephrolithiasis and renal impairment require careful patient selection [50].

### Cardio-renal-cometabolic agents: dual action and mechanisms

Some antihypertensive and metabolic agents possess urate-lowering effects that benefit patients with gout and comorbidities.

Losartan (angiotensin receptor blocker) exerts mild uricosuric effects and is especially useful in hypertensive gout patients [51].

Certain calcium channel blockers: (notably amlodipine) lower urate modestly while controlling blood pressure [52].

Fenofibrate, a lipid-lowering agent, enhances uric acid excretion *via* renal mechanisms, serving patients with concurrent hyperlipidemia and gout [51].

SGLT2 inhibitors (dapagliflozin, canagliflozin, empagliflozin) increase renal uric acid excretion and have been shown in recent trials to lower serum urate, reduce gout risk in diabetics, and provide cardiovascular and renal benefits [39].

### Biologic therapies targeting inflammatory pathways

Biologics such as anakinra (IL-1 receptor antagonist), canakinumab (monoclonal anti-IL-1β antibody), and rilonacept (IL-1 trap) directly target the IL-1β pathway, a key mediator of gouty inflammation and neutrophil recruitment [53,54].

Canakinumab has demonstrated significant efficacy in acute and refractory cases, substantially reducing flare frequency and severity, as confirmed in pivotal RCT [55]. The long-term extension data support durable benefit, though high cost and infection risk limit universal access.

Anakinra and rilonacept provide alternatives – anakinra is used off-label for acute flares, especially in complex or contraindicated cases, and rilonacept is newly FDA-approved for recurrent gout flares [56,57].

These agents exemplify translational targeting of key inflammatory mediators, allowing rapid control of flares, especially in patients intolerant of standard therapies.

### Novel and investigational agents

#### Selective xanthine oxidase inhibitors

Tigulixostat is a new, non-purine XOI that inhibits uric acid production, similar to allopurinol and febuxostat. Recent phase II/III trials demonstrate dose-dependent serum urate reduction, efficacy comparable to established agents, and good tolerability. Approved in Japan (2023), it is a promising option, especially for patients intolerant to current XOIs [45].

#### Uricase therapies

Pegloticase, a PEGylated recombinant uricase, converts uric acid to allantoin (a more soluble compound) for rapid reduction of serum urate and resolution of tophi in severe/refractory gout. Pegloticase’s use is limited by infusion reactions, anti-drug antibody development, high cost, and the need for IV administration [58].

### Emerging/Adjunct approaches

Ongoing studies are evaluating agents that target additional steps in urate metabolism or immune modulation, such as investigational uricosurics (arhalofenate, verinurad) and potential microbiome-directed approaches [59].

### Comorbidity burden and dual-action therapies in gout

Gout is increasingly recognized as a disease with substantial comorbidity burden, particularly related to cardio-renal-metabolic disorders such as hypertension, chronic kidney disease, type 2 diabetes, and dyslipidemia [60]. This comorbidity burden has significant implications for both treatment selection and long-term management. In recent years, attention has shifted toward the use of medications that address both gout and its associated comorbidities, offering a dual therapeutic benefit for patients who frequently present with complex health needs.

Several medications with primary indications in cardiovascular or metabolic disease have demonstrated urate-lowering effects or benefits for gout management. For instance, losartan, an angiotensin II receptor blocker, possesses mild uricosuric properties and can be beneficial for patients with hypertension and hyperuricemia [51,61]. Similarly, certain calcium channel blockers (notably amlodipine) may also lower serum urate and are preferred for blood pressure control in patients with gout [52]. Fenofibrate, a lipid-lowering agent, can reduce serum urate by promoting renal serum urate excretion, making it advantageous in patients with co-existing hyperlipidemia and gout [52]. More recently, SGLT2 inhibitors – used in the management of type 2 diabetes and chronic kidney disease – have been shown to lower serum urate levels and may reduce gout incidence while offering cardiovascular and renal protection [62].

The integration of such agents into the therapeutic strategies for gout reflects a comprehensive approach to patient management: not only controlling hyperuricemia and preventing gout flares, but also targeting the underlying metabolic and cardiovascular risks that are prevalent in this population. As further research clarifies the impact of these medications on gout outcomes, their use may become more prominent in personalized, comorbidity-driven gout care.

### Personalized medicine approaches: From concept to clinical practice

Recent progress in genomic and biomarker research has catalyzed a shift toward precision medicine in the management of gout, allowing for increasingly individualized and effective therapeutic strategies. Beyond broader concepts, several key genetic markers now hold tangible relevance in clinical decision-making for patients with gout.

#### HLA-B*58:01 and allopurinol hypersensitivity

A paradigm-defining example is the HLA-B58:01 allele, which is strongly associated with a significantly increased risk of allopurinol-induced severe cutaneous adverse reactions (SCAR), including Stevens-Johnson syndrome and toxic epidermal necrosis [63]. The utility of HLA-B58:01 genotyping prior to allopurinol initiation is well-documented, particularly in East Asian populations, where carriage rates are higher and risk is markedly increased [64,65]. Current guidelines, including those from the American College of Rheumatology (ACR) and European Alliance of Associations for Rheumatology (EULAR), recommend HLA-B*58:01 screening in high-risk ethnic groups before starting allopurinol therapy [66]. Identifying carriers enables clinicians to avoid allopurinol and select alternative urate-lowering therapies, thereby significantly reducing the incidence of life-threatening adverse events and exemplifying the direct benefit of genetics in everyday clinical practice.

#### Other genetic and genomic markers: toward targeted urate-lowering therapy

In addition to HLA-B*58:01, ongoing research is illuminating how common variants in genes encoding urate transporters – such as ABCG2, SLC2A9 (GLUT9), SLC22A12 (URAT1), and others – modulate serum urate levels and gout risk in different populations [67]. For example:

ABCG2 polymorphisms: Associated with both increased gout susceptibility and poorer response to certain urate-lowering therapies, particularly in young-onset disease [68].

SLC22A12 (URAT1) variants: Linked to differences in renal uric acid handling, potentially informing the choice and expected efficacy of uricosuric agents [67].

CYP2C9 and CYP2C19 variants: May influence the metabolism and toxicity of some medications used in gout management [69].

Although pharmacogenetic testing for these markers is not yet routinely integrated into gout management, such research is paving the way for treatment algorithms that optimize efficacy and minimize adverse effects based on the individual’s genetic profile.

### Combination therapies

Combining traditional therapies with emerging approaches can offer patients a more effective treatment regimen, tailored to their individual needs. By integrating biologics, novel agents, and personalized medicine strategies, clinicians can create a comprehensive treatment plan that addresses multiple aspects of gout pathology. Different classes of medications can have complementary mechanisms of action. For example, traditional urate-lowering therapies such as xanthine oxidase inhibitors (e.g., allopurinol or febuxostat) effectively reduce serum urate production, while biologics like anakinra inhibit IL-1β, targeting inflammation directly during acute gout flares [70]. By combining these therapies, patients can benefit from immediate inflammation control while simultaneously lowering serum urate levels, thereby addressing both facets of the disease [71]. Some patients may demonstrate suboptimal responses or develop resistance to monotherapy. Combination therapies help to address the potential limitations of individual treatments and can enhance overall therapeutic efficacy. For instance, if a patient does not achieve sufficient serum urate control with allopurinol alone, adding a uricosuric agent like probenecid could help improve results [72]. Individual patient factors, such as comorbidities, genetic predispositions, and medication tolerability, require a tailored approach to treatment. For example, patients with renal impairment may necessitate different combinations compared to those without.

#### Examples of combination therapy strategies

Incorporating uricosuric agents, such as probenecid or lesinurad, alongside traditional therapies can enhance the excretion of serum urate, thereby supporting xanthine oxidase inhibitors in achieving targeted serum urate levels [72]. This synergistic effect could be particularly beneficial for patients with decreased renal clearance of serum urate or those who experience inadequate responses to monotherapy.

For patients suffering from diabetes alongside gout, incorporating metformin into the therapeutic regimen may provide dual benefits. This combination can help manage blood glucose levels while also effectively controlling serum urate levels [73].

Recent research suggests the gut microbiome may influence serum urate metabolism and inflammation in gout patients. Investigating the potential of probiotics or dietary interventions designed to modulate the microbiome could lead to novel adjunct therapies [74].

### Treat-to-target serum urate: Controversy and ongoing research

The optimal approach to serum urate monitoring and management in gout remains an area of active debate. International guidelines generally recommend a treat-to-target strategy – that is, titrating urate-lowering therapy (ULT) to achieve and maintain a serum urate of <6 mg/dl (<360 μmol/l), or even lower in severe cases [EULAR,ACR]. However, the evidence base for this strategy, especially in terms of hard outcomes such as flare reduction, tophus resolution, and comorbidity prevention, is still being established [75].

Some controversies stem from concerns about over-treatment, polypharmacy, patient adherence, and the lack of definitive randomized controlled trial (RCT) data confirming clear superiority of treat-to-target over fixed-dose approaches. To address this, several large clinical trials are currently underway or have recently reported results:

- TICOG (Tight Control of Gout): Compares a treat-to-target urate-lowering strategy versus routine care [76].
- TRUST: Assesses the effect of a treat-to-target serum urate approach in older adults with gout [77].
- NOR-Gout: Explores whether intensive urate-lowering improves outcomes vs. less stringent management [77].
- Gout Treatment Strategy Overture Trial: A prospective study examining treat-to-target versus symptom-driven therapy.
- UK Gout Management Trial: Aims to determine if a treat-to-target approach yields superior long-term results.

Results from these studies are expected to clarify whether aiming for strict serum urate targets translates into tangible clinical benefits for patients and could influence future guideline recommendations. Until then, shared decision-making that balances guideline recommendations, patient preferences, and comorbidity risk remains the cornerstone of modern gout management.

### Clinical considerations and challenges

Tailoring treatment strategies to accommodate individual patient needs is critical in gout management. Factors such as age, comorbidities – including renal insufficiency, cardiovascular disease, diabetes, and obesity – and patient preferences can guide the choice and combination of therapies. For example, allopurinol is often the first-line urate-lowering therapy, but may require dose adjustment in patients with renal impairment, while febuxostat can be considered in those with mild to moderate chronic kidney disease, though its cardiovascular safety profile should be considered [78]. Losartan or calcium channel blockers may be preferred as antihypertensive agents for gout patients due to their modest uricosuric effects [42].

Awareness of potential side effects from combination therapies is essential. For instance, pegloticase, though effective in refractory cases, may cause infusion reactions and requires close monitoring, while the combination of xanthine oxidase inhibitors with uricosurics like probenecid increases the risk for nephrolithiasis and other adverse effects [79,80]. Increased polypharmacy can complicate patient care, making adverse drug reactions and drug-drug interactions more likely. Clinicians should educate patients on recognizing side effects early and maintain open communication regarding any symptoms or concerns. Implementing routine follow-ups and monitoring (e.g., regular serum urate and renal function measurements) can help clinicians promptly address emerging issues, enhancing therapeutic outcomes. Disparities in healthcare access remain a key barrier to effective gout management, especially in low-resource settings or where combination therapies and newer biologics are costly or unavailable. For instance, medications like canakinumab and pegloticase may be out of reach for many patients due to limited insurance coverage [81]. Patients from low-income backgrounds may face treatment gaps and suboptimal disease control. Addressing these inequalities requires ongoing advocacy for improved health policy and broader access to essential medications. Community outreach and partnerships with local pharmacies may help improve access to care for underserved populations.

### Multidisciplinary approaches and implementation barriers

The effective management of gout often necessitates a multidisciplinary team, including rheumatologists, primary care physicians, pharmacists, nurses, and dietitians, each providing distinct expertise across the care continuum [82,83]. Rheumatologists can optimize pharmacologic regimens, especially in complex or refractory cases, while primary care providers frequently serve as the first point of contact and are vital for ongoing monitoring and management [84]. Pharmacists play a pivotal role in patient education, medication reconciliation, identifying drug-drug interactions, and supporting adherence to urate-lowering therapy. Dietitians address nutritional counseling, providing practical guidance on healthy dietary patterns and lifestyle modifications tailored to patient preferences and comorbidities.

Studies have shown that coordinated, multidisciplinary approaches can improve serum urate target attainment, enhance patient satisfaction, and reduce flares [85]. Nurse-led or pharmacist-led interventions may further enhance adherence, provide regular patient contact, and deliver ongoing education [86]. However, real-world implementation faces substantial barriers: limited resources, fragmented communication across specialties, inadequate referral pathways, and patient factors such as health literacy or complex social needs. In some healthcare systems, dietitian and pharmacist support may be difficult to access due to cost constraints or lack of formal, supported care pathways.

Addressing these barriers requires ongoing system-level changes: integrated electronic care records, formalized care pathways, staff training, and efforts to reduce cost and access inequities. Engaging patients and families directly in self-management planning is also critical to the success of multidisciplinary care.

Evaluating the long-term efficacy of combination therapies is essential for optimizing gout management. While immediate responses to treatment are crucial, understanding the chronic implications of therapy is equally important. Clinicians should implement systems for long-term follow-up that track patient outcomes over time, which will inform adjustments to treatment regimens as needed. Engaging patients in discussions about their long-term goals and monitoring their progress toward those goals can enhance adherence and improve overall treatment satisfaction.

## Treatment challenges and unmet needs

Despite significant progress in the understanding and management of gouty arthritis, several challenges persist, necessitating ongoing research and innovative solutions to enhance patient outcomes.

### Adverse effects of current therapies

While most current pharmacological treatments for gout are generally effective, in some patients, adverse effects can affect compliance and overall tolerability. Potential adverse effects of NSAIDs include gastrointestinal complications, renal toxicity, or cardiovascular events [87]. Colchicine may cause gastrointestinal adverse effects, while prolonged administration of corticosteroids can result in the loss of bone density, among other systemic adverse effects [88]. Identifying alternative treatments with fewer side effects or developing strategies to mitigate these adverse effects is of critical importance for optimizing the long-term safety and tolerability of gout therapies.

### Patient adherence and compliance

Effective gout management is highly dependent on patient adherence to long-term urate-lowering therapy (ULT) and lifestyle recommendations. However, optimal adherence remains challenging due to the chronic nature of gout, the need for daily medication even when asymptomatic, and concerns about adverse effects or perceived lack of necessity [89]. Studies indicate that up to 50 % of gout patients discontinue ULT within one year, with non-adherence leading to increased risk of recurrent flares and joint damage [90].

**Fig. 3:**
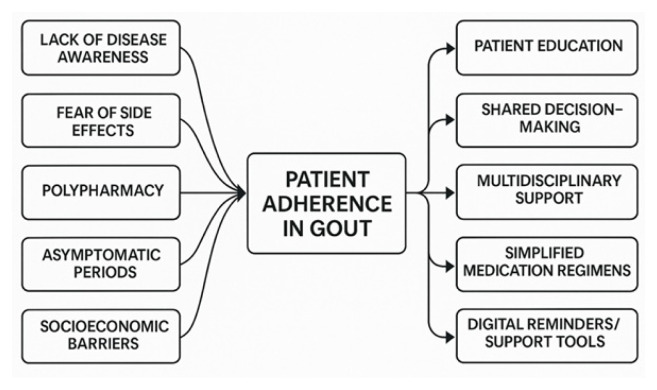
Barriers and solutions to patient adherence in gout management.

Key barriers to adherence include insufficient patient understanding of gout as a chronic disease, fears about medication side effects, and a lack of regular follow-up or communication with healthcare professionals [91]. To overcome these challenges, clinicians should provide clear education about the role and expected course of ULT, address misconceptions and concerns, and encourage open dialogue regarding adverse effects or difficulties with medications. Regular nurse- or pharmacist-led follow-ups, use of reminders or digital health tools, and patient-centered care plans have all been shown to improve adherence [92]. Furthermore, development of new formulations or delivery methods, such as once-daily oral agents or long-acting injectables, can enhance convenience and acceptability, further supporting compliance (Fig. 3).

### Addressing gaps in current understanding

Despite significant advances, key aspects of the pathogenesis of gouty arthritis remain unclear. The complex interplay of genetic, environmental, and lifestyle factors requires further study. In particular, the mechanisms underlying the initiation and resolution of gout flares are not fully understood, hindering the development of more targeted therapies. Important gaps also include understanding the heterogeneity of gout, determining why some individuals experience more severe disease or complications, and developing personalized treatment strategies. Additionally, challenges persist in the management of asymptomatic hyperuricemia, long-term prevention of joint damage, and optimal control of comorbidities. Addressing implementation barriers – such as patient adherence, health literacy, and equitable access to advanced therapies – remains a priority for future research (Fig. 3) [93,94].

## Future directions in Gouty Arthritis research

The future landscape of research into gouty arthritis holds the prospect of transformative breakthroughs. Further studies are perched on a newfound interest in exploring innovative approaches to improve diagnosis, treatment monitoring, and the development of new therapeutic interventions.

### Advancements in imaging techniques

The incorporation of advanced imaging techniques is revolutionizing the approach to gout management, enabling clinicians to tailor treatment strategies more effectively. Positron Emission Tomography (PET) is another emerging imaging modality that holds promise for gout diagnosis. PET can provide metabolic information about inflammation in affected joints, allowing for a more comprehensive understanding of the disease’s pathophysiology. Future research could explore the correlation between PET findings and clinical outcomes, potentially establishing PET as a valuable tool in monitoring treatment response and disease activity [18].

Optical Coherence Tomography (OCT) is also gaining attention in the realm of musculoskeletal imaging [95]. With its ability to provide high-resolution cross-sectional images of joint structures, OCT could facilitate the early detection of subclinical gout and the assessment of intra-articular changes. Investigations into the integration of OCT with other imaging modalities may yield synergistic benefits, enhancing the overall diagnostic capabilities for gout [96].

Moreover, the development of portable imaging devices is set to transform the accessibility of gout diagnosis. Handheld ultrasound devices, for example, can be utilized in primary care settings, allowing for immediate assessment and reducing the need for referrals to specialists. Research aimed at evaluating the effectiveness of these portable devices in diverse populations could help bridge gaps in care, particularly in underserved regions where access to advanced imaging is limited.

As these imaging technologies continue to evolve, the focus on interdisciplinary collaboration will be crucial. Radiologists, rheumatologists, and primary care physicians must work together to develop standardized protocols and guidelines for the use of these advanced imaging techniques in clinical practice. This collaborative approach can ensure that imaging findings are integrated into a holistic treatment plan, ultimately improving patient outcomes.

### Biomarkers for treatment response

In addition to the previously mentioned markers, microRNAs (miRNAs) are emerging as a promising class of biomarkers in gout research. These small non-coding RNA molecules play a pivotal role in regulating gene expression and may be involved in the inflammatory processes associated with gout flares. Studies investigating profiles of specific miRNAs in patients with gout could reveal patterns that distinguish between active and inactive disease states, allowing for better prognostication of flare occurrences and response to treatments [97].

Furthermore, serum urate oxidation products like oxypurines and allantoin are garnering attention as potential biomarkers [98]. Their levels could provide insight into oxidative stress and the overall metabolic state of patients with gout. Analyzing the relationship between these products and patient outcomes may yield critical information that assists in fine-tuning urate-lowering therapies [99].

Utilizing a multi-biomarker approach may provide a more nuanced understanding of disease activity and treatment response, enhancing personalization in gout management. For instance, a panel that includes inflammatory markers, metabolic indicators, and genetic variants could provide a holistic view of the patient’s condition, guiding clinicians in tailoring therapies that align with the individual’s unique profile.

### Targeting novel pathways

As research continues to reveal the complex biology underlying gout, targeting novel pathways offers the potential for innovative treatment options that address both the acute and chronic phases of the disease [100]. Metalloproteinase inhibitors represent another therapeutic avenue that warrants exploration. These agents could help modulate cartilage degradation and joint damage by inhibiting enzymes responsible for the breakdown of extracellular matrix components in arthritic conditions exacerbated by gout [101]. Future studies could assess their role in preserving joint function and improving patient outcomes.

Additionally, pro-resolving mediators (PRMs), derived from omega-3 fatty acids, may provide a novel strategy to resolve inflammation [102]. These endogenous substances facilitate the resolution of inflammatory processes and may counteract the chronic inflammation typical of gout. Investigations into the therapeutic application of PRMs could highlight their potential to not only mitigate acute flares but also promote longer-term joint health [103].

The use of antioxidants is also emerging as a potential approach to manage oxidative stress and inflammation associated with gout. Compounds such as resveratrol and curcumin, known for their anti-inflammatory and antioxidant properties, could be studied for their efficacy in clinical settings [104,105]. A focus on how these agents might supplement existing therapies by enhancing overall treatment efficacy could pave the way for combination strategies that target multiple pathways concurrently.

Gene therapy is another frontier in the treatment of gout, with the potential to address the root causes of hyperuricemia and joint inflammation [106]. While still in the early stages, the application of gene-editing technologies may hold promise for creating long-lasting therapeutic options.

Collaboration across disciplines will be key to advancing our understanding of these novel pathways. Research teams comprising molecular biologists, pharmacologists, and clinicians can identify biomarkers to monitor treatment responses effectively, ensuring clinical studies are well-informed and targeted. By embracing a holistic view that encompasses not just symptom management but also underlying pathophysiological mechanisms, the field of gout therapy can evolve significantly.

### Exploring new treatment strategies

The management of gout is evolving, with emerging strategies focusing on precision medicine, education, and comprehensive care. Precision medicine involves tailoring treatment plans to individual patient characteristics, such as genetic profiles, comorbidities, and lifestyle factors [107]. This approach allows clinicians to enhance therapeutic outcomes by selecting the most suitable interventions for each patient. For instance, certain genetic polymorphisms, such as those in the SLC2A9 and ABCG2 genes, can influence patient responses to urate-lowering therapies [108]. Clinical trials have shown that genetic screening may guide treatment decisions, enabling a more personalized approach to managing gout [109].

Novel delivery systems for existing medications, such as transdermal patches or inhalable formulations of urate-lowering agents, may enhance bioavailability and patient compliance. Clinical trials investigating these alternative delivery methods can provide valuable insights into their efficacy and safety. Community health initiatives, including awareness and screening programs, can help identify at-risk populations and encourage early intervention. Collaborating with local health organizations to provide education and support can facilitate timely diagnosis and treatment, reducing the incidence of acute flares.

## Conclusions

In summarizing the comprehensive review on gouty arthritis, we reflect upon key findings, implications for clinical practice, and the need for further research in order to continue our understanding and management of this complex rheumatic condition.

This review has been a journey into the many dimensions of gouty arthritis, from its complicated pathophysiology to the various different methods of treatments and therapies that continue to evolve. From the deposition of crystals secondary to hyperuricemia to the detailed mechanisms of action of pharmacological agents, with the emergence of new therapies, has come a newer understanding of gout. These insights gained from this exploration have immediate implications for clinical practice. Clinicians should consider gout, a condition resulting not only from crystal deposition but one that involves a dynamic interaction between genetic predisposition, immune responses, and lifestyle factors. Treatment needs to be individualized based on specific patient profiles, with the goal of fostering better patient education to improve compliance and considering the potential impact of adverse effects on long-term therapy.

The integration of advanced imaging methods, biomarkers, and personalized medicine approaches into everyday clinical practice may significantly change the diagnosis, monitoring, and treatment of gout. Clinicians are urged to stay abreast of emerging research and incorporate evolving strategies into their practices to improve patient outcomes and enhance the quality of gout management.

While significant strides have been made, overcoming the complexities of gouty arthritis will be far from over. Adverse effects, patient adherence, and knowledge gaps invite the research community to press deeper. Future studies need to use new directions, leveraging new imaging advances, establishing strong biomarkers for treatment response, and discovering novel targets of therapy.

We call for collaboration by researchers, health professionals, and stakeholders in a coordinated effort to drive innovation and translate discovery into clinical practice. By fostering an environment of continuous inquiry and innovation, we can collectively propel the field forward and offer hope to the millions affected by gouty arthritis.

In conclusion, this review is intended to provide clinicians and researchers with a practical synthesis of recent advances in the management of gouty arthritis. The practical implications outlined herein are designed to support evidence-based decision-making, facilitate the adoption of new therapies, and inform future gout research priorities.

## Figures and Tables

**Table 1:** Overview of current treatment approaches.

Treatment Category	Examples	Mechanism of Action
**Pharmacological Interventions**	NSAIDs (Indomethacin, Naproxen)	Inhibition of cyclooxygenase enzymes, reducing inflammation
	Colchicine	Disruption of microtubule formation, inhibiting inflammation
	Corticosteroids (Intra-articular, systemic)	Anti-inflammatory effects, rapid relief during acute flares
	Xanthine Oxidase Inhibitors (Allopurinol, Febuxostat)	Reduction of serum urate production
	Uricosuric Agents (Probenecid)	Enhancement of renal excretion of serum urate
**Lifestyle Modifications**	Dietary Changes (Low-purine diet: avoid organ meats, certain seafood)	Reduction of purine-rich foods, supporting serum urate control
	Hydration (Increased fluid intake)	Promoting serum urate excretion
	Weight Management	Addressing obesity as a risk factor for gout
	Alcohol Consumption	Avoidance of alcohol

**Table 2:** Emerging therapeutic strategies in gout: key agents, evidence, and practical considerations.

Therapeutic Approach	Examples	Mechanism of Action	Key Trials/Systematic Review	Efficacy	Adverse Effects	Approval/Availability	Cost/Other Factors
*Biologics targeting IL-1β*	Anakinra, Canakinumab, Rilonacept	Inhibit IL-1β, reducing neutrophilic inflammation	Saag [35] Jena [36] Maher [37]	High efficacy in acute, refractory, or CI to standard ULT	Infection, injection site reaction, rare neutropenia	Canakinumab, Rilonacept: FDA/EMA Anakinra: off-label	High cost; limited insurance for chronic use
*Uricase/PEGylated Uricase*	Pegloticase	Converts uric acid to allantoin (more soluble compound)	Botson [38]	High efficacy in refractory, tophaceous gout	Infusion reactions, anaphylaxis, anti-drug antibody formation	FDA approved (US)	Very high cost; IV infusions; for severe/refractory disease
*SGLT2 Inhibitors*	Dapagliflozin, Canagliflozin, Empagliflozin	Increases renal uric acid excretion *via* SGLT2 inhibition	Yang [39] Yokose [39] Tesfaye [40]	Lowers serum urate, reduced gout risk in Diabetics	Genital infections, dehydration, euglycemic ketoacidosis	Approved for DM/CKD/HF (global)	Oral; cost varies by region; benefit for DM, CVD, CKD
*Cardio-renal-cometabolic agents*	Losartan, Amlodipine, Fenofibrate	Uricosuric, uric acid lowering or excretion	Zhou [41] Jiao [42] Kaufmann [43]	Mild-moderate urate lowering (adjunctive)	Hypotension, renal function, myopathy (fibrate)	Widely available, off-label	Generic for most; highly accessible

*Personalized/genomic medicine*	Genotyping (ABCG2, SLC2A9, URAT1, etc.)	Treatment tailored by urate transporter gene variants	Azwan [44]	Guides agent choice, risk stratification	Minimal direct adverse effects (from testing)	Not routinely available still	Cost, access, insurance coverage varies
*Selective Xanthine Oxidase Inhibitor*	Tigulixostat	Inhibits xanthine oxidase, lowering uric acid production	Terkeltaub [45] Saag [46] Hsu [47]	Comparable to febuxostat in urate lowering; effective in hyperuricemia	Mild LFT elevation, GI symptoms; well tolerated in trials	Approved in Japan (2023); not FDA-approved yet	Oral dosing; potential alternative for allopurinol-intolerant patients

CI: Contraindication; DM: Diabetes Mellitus; CVD: Cardiovascular disease; CKD: Chronic Kidney Disease; FDA: US Food and Drug Administration; EMA: European Medicines Agency; IV: Intravenous; ULT: Urate-lowering therapy.
